# X-linked diseases: susceptible females

**DOI:** 10.1038/s41436-020-0779-4

**Published:** 2020-04-14

**Authors:** Barbara R. Migeon

**Affiliations:** Departments of Genetic Medicine and Pediatrics, The Johns Hopkins Medical Institutions, Baltimore, MD USA

## Abstract

The role of X-inactivation is often ignored as a prime cause of sex differences in disease. Yet, the way males and females express their X-linked genes has a major role in the dissimilar phenotypes that underlie many rare and common disorders, such as intellectual deficiency, epilepsy, congenital abnormalities, and diseases of the heart, blood, skin, muscle, and bones. Summarized here are many examples of the different presentations in males and females. Other data include reasons why women are often protected from the deleterious variants carried on their X chromosome, and the factors that render women susceptible in some instances.

## INTRODUCTION

Sex differences in human disease are usually attributed to sex specific life experiences, and sex hormones that influence the function of susceptible genes throughout the genome.^1–5^ Such factors do account for some dissimilarities. However, a major cause of sex-determined expression of disease has to do with differences in how males and females transcribe their gene-rich human X chromosomes, which is often underappreciated as a cause of sex differences in disease.^6^ Males are the usual ones affected by X-linked pathogenic variants.^6^ Females are biologically superior; a female usually has no disease, or much less severe disease than the male with the same variant, unless she is homozygous for the deleterious allele, or it is lethal for males.

The X chromosome carries 867 known protein coding genes.^7^ Clearly, pathogenic variants that induce complete loss of function may be lethal to fetuses of both sexes; however, a number of these pathogenic variants—less severe, or occurring in less-essential genes—cause at least 533 X-linked diseases^8^ that affect males more severely.^8^ Rather than influencing sexual development, most of these genes play a role in nonreproductive human tissues, including brain, bone, blood, ears, heart, liver, kidney, retina, skin, and teeth.

Table 1 provides data about a substantial number of X-linked disorders obtained in large part from OMIM^8^ that confirm the lesser susceptibility of females. The table is not all-inclusive, but it provides enough data to show the greater severity of these diseases in males, and to illustrate why some, but not all, females with the same X-linked deleterious allele are protected from its effects. This paper is motivated by the question: When so many women are protected from manifesting severe X-linked diseases, why are some of them susceptible?

**Table. 1:** Effect of X-inactivation (XI) on phenotype and cell selection in X-linked disorders.

X-linked disease	X Map	Gene	OMIM	Phenotype: males	Phenotype: females	Cell selection	Tissue	XI skewing assay
**Aarskog–Scott syndrome** **allelic with XLMR 16**	**Xp11.2** **54,445,453**	***FGD1*** **premature termination;** **truncation**	305400	Faciogenital dysplasia (ocular hypertelorism, shawl scrotum) with attention deficit hyperactivity, also XLID	Subtle features as widows peak or short stature Skewing toward mutant increases severity (i.e., translocation)	ND		
**XLMR 16**	**Xp11.22** **54,445,453**	***FGD1*** **missense**	305400	ID only	No affected females	ND		
**Acrogigantism X-LAG** **allelic with X-linked immunodeficiency with hyper IGM**	**Xq26.3** **136,648,176**	***CD40LG*** 300386 **microduplication**	300942	Microduplications: only mosaic males are cited, so may be lethal in most males; mosaic males have acrogigantism, but no immunodeficiency	Females are like mosaic males; acrogiantism, but no immunodeficiency Elevated growth hormone & prolactin	Microduplications have random XI Affected females may not be skewed until older age	Lymphocytes	*AR*
**Immunodeficiency with hyper IGM (HIGM) Immunodeficiency 3**	**Xq26.3** **136,648,176**	***CD40LG*** **(CD40 ligand on T cells)** **variants**	308230	Decreased IgG, IgA, & IgE; susceptibility to opportunistic bacterial diseases, leading to liver disease; most have severe infections & shortened life spans	Heterozygotes have normal levels of IgG, IgA, IgM, & IgE	Not convincing as reports conflict; it seems that half normal is enough to protect females	T and B cells & fibroblasts	
Adrenoleukodystrophy	Xq28 153,724,850	*ABCD1*	300100	Demyelinization of brain, spinal cord, & adrenals. Often death in first decade	Adrenomyeloneuropathy; spastic paraplegia with age	Yes, gradual. Favors mutant allele	WBC, RBC, skin fibroblasts clones	*G6PD* & fatty acids
Alport syndrome	Xq22.3 108,439,837	*COL4A5*	301050	End stage renal disease; hearing loss; ocular malformations	Milder renal disease Severity related to skewing	Severity related to skewing toward mutant allele	WBC Kidney glomeruli	*HPRT* & *PGK.* *COL45A* Immunolabel
Amelogenesis imperfecta	Xp22.2 11,293,412	*AMELX*	301200	Hypoplastic amelogenesis imperfecta; Mottled teeth (fluoride independent); Homogeneous pattern of abnormality AMELY expressed at 10% activity of AMELX	Vertically grooved teeth; variable depending on skew of XI	One homozygous female was affected like her hemizygous father, and more severe than her heterozygous mother; mother 25% skewed toward mutant, reflected in degree of grooving	WBC	*AR*
**Androgen insensitivity**	**Xq12** **67,544,020**	**AR** **loss of function hypomorphic**	300068	Feminization or hypospadias and micropenis	No affected females	ND Not severe (two clonal populations, but lower binding in heterozygotes)	Skin fibroblast clones, both normal and mutant present	Androgen binding
**Kennedy spinal bulbar & muscular atrophy**	**Xq12** **67,544,020**	**AR** **trinucleotide repeat expansion**	313200	36–62 repeats in males; onset: 3rd–5th decade; slowly progressive muscle atrophy; progressive decrease in sperm production	Affected, if homozygous, but less affected because of higher androgens in males; heterozygotes usually normal, but some muscle cramps	ND		
**ATRX syndrome** **ɑ-thalessemia/ID syndrome**	**Xq21.1** **77,504,877**	***ATRX*** **300032**	301040	ID, thalassemia, genital abnormalities attributable to variants in PHD domain	Hemoglobin H inclusions Mild retardation, usually unaffected	Yes, severe toward wild type allele	WBC, buccal smear	*AR*
ɑ**-thalessemia Myelodysplastic syndrome**	**Xq21.1** **77,504,877**	***ATRX***	300448	Severe variant reducing activity to 3–4% normal	No affected females	Yes, severe toward wild type allele	WBC	*AR*
**MR-hypotonic facies syndrome**	**Xq21.1** **77,504,877**	***ATRX***	309580	Not so severe variants or those in the helical domain No genital abnormalities	No affected females	Yes, severe toward wild type allele	WBC	*AR*
Barth syndrome	Xq28 154,411,517	*TAZ*	302060	Idiopathic cardiomyopathy Methylglutaconic aciduria Abnormal mitochondria Death in childhood	No affected females	Yes, severe toward wild type allele	WBC, fibroblasts	*AR* in obligate heterozygotes
Borjeson–Forssman–Lehmann syndrome	Xq26.2-3 134,373,311	*PHF6*	301900	ID, obesity, hypogonadism, epilepsy, facial dysmorphism	Mild ID 11 females with de novo variants have Coffin–Siris phenotype all skewed 100% toward mutant allele, female with 70% skewing had milder symptoms	Yes, severe toward wild type allele If unskewed, then manifesting at least a little If skewed severely toward mutant, then severe disease	WBC	*FMR1, AR PGK1*
Bruton agammaglobulinemia	Xq22.1 101,349,446	*BTK*	300300	B-cell deficiency; boys lack circulating B cells; they are overcome by bacterial infections	No affected females	Yes, severe >95%	B cells	*AR*
**Cataract 40** **Allelic with Nance–Horan syndrome**	**Xp22.1-22.2** **17, 375,199**	***NHS*** **Lack of NHS leads to NH syndrome; milder variants give cataracts**	302200	Congenital cataracts with severe visual impairment & microcornea Associated with triplication of the locus	Normal vision but develop cataracts in their 40s	ND		
**Nance–Horan Syndrome**	**Xp22** **17, 375,199**	***NHS***	302350	Congenital cataract leading to profound vision loss; dysmorphic features and malformed teeth Microcornea, microphthalmia, and mild or moderate ID	Slightly reduced vision	ND		
Charcot–Marie–Tooth CMTX1	Xq13.1 71,215,211	*GJB1*	302800 304040	Sensory & peripheral neuropathies	Milder	No	WBC	*AR*
**Charcot–Marie–Tooth** **CMTX5** **Allelic with DFNX1** **& PRPS1 related gout**	**Xq22.3** **107,628,423**	***PRPS1***	311070	Optic atrophy, polyneuropathy, & deafness	Milder	No skewing determines severity	WBC	*AR*
Charcot–Marie–Tooth CMTX6	Xp22.11 24,465,226	*PDK3*	300905	1 three-generation family Males more severe than females Foot deformities, abnormal gait muscle weakness, sensory abnormalities	Subtle features such as hand tremor with age	ND		
Christianson syndrome	Xq26.3 135,974,595	*SLC9A6* (*NHE6*)	300231	Profound ID; mute; developmental regression; impaired ocular movements Epilepsy; microcephaly; cerebellar and brain stem atrophy	Milder Psychiatric disorders Study of 20 female heterozygotes shows deficit in at least one neurocognitive domain (ID 20%, learning differences 31%, speech delays 30%, & ADHD 20%); atypical parkinsonism, with age	ND		
**CHILD syndrome**	**Xq28** **152 830, 966**	***NSDHL*** **loss of function** **missense & nonsense**	308050	Fetal lethal	Hemidysplasia with Unilateral ichthyosis	Yes, (in mice)	Brain, skin, liver of *Bare Patches* mice	*NSDHL* activity
**CK syndrome** **Analogous to bare patches in mice** **Allelic with CHILD syndrome**	**Xq28** **152 830, 966**	***NSDHL*** **300275** **hypomorphic variant**	300831	ID plus neonatal seizures Only males affected; Defect in cholesterol synthesis	Heterozygotes not affected	ND		
Chondrodysplasia punctata 1	Xp22.23 2,934,631	*ARSE* sometime small chromosomal deletions	302950	ID, bone defects; short stature; epiphyseal stippling	Milder symptoms	No Mild cases not affected	WBC & fetal tissues	*AR*
**Chondrodysplasia punctata 2** **(Conradi–Hunermann syndrome) CDPX2** **Allelic with Mend syndrome**	**Xp11.23** **48,521,807**	***EPB*** **Emopamil binding protein**	302960	Fetal lethal; Facial skin and skeletal dysplasia Only mosaic males survive	Bilateral ichthyosis Short stature Epiphyseal stippling Hair and skin defects Occasionally severe due to skewed XI	No	WBC	*AR*
**Conradi–Hunermann–Happle syndrome CDPX2**	**Xp11.23** **48,521,807**	***EBP***	302960	Mosaic grandfather (50%) Short stature	Mother: short stature; Mosaic skin defect Fetus: severe bone abnormalities; no skin rash	Random XI in blood of both fetus & mother (not shown) Perhaps skewed in affected tissues	WBC	*AR* splicing pathogenic variant in EBP causing extreme familial variability
**MEND syndrome**	**Xp11.23** **48,521,807** **missense**	***EBP*** **Hypomorphic variant**	300960	Nonmosaic; ID; short, scoliosis; abnormal digits; cataracts and dermatitis	Heterozygotes are usually unaffected	ND		
Congenital disorder of glycosylation CDG2M	Xp11.23 48,903,182	*SLC35A2* UDP galactose transporter Loss of function	300896	All affected males are mosaics	Females are affected with infantile epileptic encephalopathy	Affected females with truncating variants are highly skewed toward wild type; one female with de novo splice site variant had random XI	WBC	*AR*
**Chronic granulomatous disease**	**Xp21.1-11.4** **37,780, 016**	***CYBB*** **Cytochrome B Beta subunit**	306400	Severe bacterial infections	Discoid lupus Rare, severely affected female due to skewing	No	WBC, buccal	*AR*
**CGD (large study of 93 females only)**	**Xp21.1-11.4** **37,780, 016**	***CYBB***	306400	Not applicable	Milder symptoms associated with higher dehydrorhodamine oxidation (DHR); low DHR associated with manifestations No progressive skewing over time	Severe carriers had low DHR Sisters & twins highly correlated, but not with mothers	WBC	%DHR+
**Immunodeficiency 34**	**Xp21.1-11.4** **37,780, 016**	***CYBB*** **missense & nonsense variant**	300645	Severe mycobacterial infections (some TB)	Rare female	ND		
**Coffin–Lowry** **Allelic to XLMR 19**	**Xp22.12** **20,149,910**	***RPS6KA3*** **(RSK2)** **Small deletions & small duplication** **Missense variants**	303600	ID; short stature; abnormal facies, gait, & fingers; microcephaly	Milder ID than male	Yes, all seem to have significant skewing; direction not clear save for 2 mothers with predominant wild type cells	WBC	*AR RSK2*
**X-linked mental retardation 19**	**Xp22.12** **20,149,910**	***RPS6KA3*** **(RSK2)** **Hypomorphic**	300854	Moderate ID with no other anomalies	Milder nonsyndromic ID	ND		
Coagulation factor 8	Xq28 154,835,787	F8	306700	Severe <1%, moderate 2–6% or mild 6–30% residual activity	Most heterozygotes have 50% so are clinically normal; affected if homozygous mutants or if XI skewed	No, but skewing causes manifestations. Familial skewing in manifesting heterozygotes	WBC	*AR*
Coagulation factor 9	Xq27.1 139,530,719	F9	300746	Affected are mainly males	Affected females usually have skewed X-inactivation or are homozygous	No, but severe skewing responsible for manifesting heterozygotes	WBC	*AR*
**Cornelia de Lange syndrome** **5% cases attributed to** ***SMC1A*** **(Cornelia de Lange, 2)**	**Xp11.22** **53,374,148**	***SMC1A*** **Missense**	300590	More severe and fetal lethal ID, facial dysmorphisms, seizures, limb abnormalities	Most of the affected are females ID, poor growth, microcephaly, dysgenesis of corpus callosum	Escape gene ND		
**Cornelia de Lange, 2**	**Xp11.22** **53,374,148**	***SMC1A*** **Truncating variants**	300040	Fetal lethal	Infantile epilepsy	ND		
**Cornelia** **de Lange** **5** **Allelic with XLMR Wilson–Turner**	**Xq13.2** **72,329,515**	***HDAC8***	300882	Facial dysmorphism; ID; multiple congenital abnormalities	Milder	Extreme, with mutant allele inactive	WBC	*AR*
**XLMR Wilson–Turner** **Allelic with Cornelia de Lange 5**	**Xq13.2** **72,329,515**	***HDAC8*** **Point variants**	300269	ID, microcephaly Craniofacial deformities	Milder	Yes, extreme	WBC	
Craniofrontonasal syndrome	Xq13.1 68,828,996	*EFNB1* Heterozygous loss of function	304110	Hypertelorism	Craniosynostosis; craniofacial asymmetry; hypertelorism; frontonasal dysplasia; skeletal abnormalities	No, variant produces cellular interference	Blood, cranioperiosteum	*AR* immunochemistry
Creatine transporter defect	Xq28 153,687,925	*SLC6A8*	300352	ID, speech delay, seizures	Milder	No	Skin fibroblasts, blood, hair roots	*AR*
Danon disease	Xq24 120,426,147	*LAMP2*	300257	ID, cardiomyopathy, skeletal muscle weakness	Later onset	Discordant identical twins; complete skew for affected Skew responsible for heterozygous phenotype	IPS T cells	*AR*
**Deafness X-linked 1** **Allelic with Charcot–Marie–Tooth 5**	**Xq22.3** **107,628,423**	***PRPS1***	304500	Phenotypic spectrum with Charcot–Marie–Tooth Males more severe than females	Females have mild high pitch hearing loss	ND		
Deafness, X-linked 4	Xp22.12 21,705,971	*SMPX*	300066	Nonsyndromic postlingual hearing loss Earlier onset in males 2–10 years (mean 3.3 years)	Onset 3–48 years (mean 28.8)	XI skewing influences phenotype	WBC	*AR*
**Dent disease1**	**Xp11.23** **49,922,595**	***CLCN5***	300009	Nephrolithiasis Proteinuria Hypophosphotemic rickets	Less severe; rare hypercalciuria; almost never chronic renal disease	First cases show 1/1 and 2/4 cases of extreme skewing toward mutant in affected females	WBC & urine sediment	*AR* and deep sequencing
**Dent disease 2**	**Xq26.1** **129,540,258**	***OCRL1*** **Mild variant of Lowe syndrome**	300535	Proteinuria; Hypercalcemia; Nephrocalcinosis. No renal tubular acidosis,	Heterozygotes not affected	ND		
**Dent disease 2 Digenic**	**Xp11.23**, **49,922,595** **Xq26.1** **129,540,258**	***CLCN5*, OCRL1** **(digenic)**	300009 300535	Abnormal facies, ocular abnormalities, rickets, delayed growth	Heterozygotes not affected	Yes, severe	WBC	mRNA
Diabetes insipidus (nephrogenic)	Xq28 159,902,624	*AVPR2*	304800	90% are X-linked (10% are autosomal); inability to concentrate urine; unresponsive to antidiuretic hormone	Heterozygotes not affected	Asymptomatic heterozygotes have random XI (4 heterozygotes normal with 50–60% XI) Skewing toward mutant leads to disease	WBC	*AR*
Dyskeratosis congenita	Xq28 154,762,741	*DKC1*	305000	Defective telomeres; premature aging; bone marrow failure	None or milder	Yes, extreme	WBC, buccal multiple tissues	*AR*
**Dystonia parkinsonism (XDP)** **Filipino type**	**Xq13.1** **71,366,219**	***TAF1*** **Retrotransposon insert**	314250	Adult onset dystonia and symptoms of Parkinson disease	Most heterozygotes not affected; a few affected have mild dystonia, later onset manifestors said to have XI skewed toward mutant, but no studies documented	ND		
**XLID 33**	**Xq13.1** **71,366,219**	***TAF1*** **Missense variants**	300966	12 boys (9 families); global delay; syndromic ID hypotonia; facial dysmorphism; microcephaly sacral caudal remnant	Heterozygotes not affected	Yes, 100% skewing toward normal allele	WBC	*AR* &* RP2* (only WT allele in RNA confirmed by PCR)
**Ectodermal dysplasia and immune deficiency**	**Xq28** **154,542,211**	***IKBKG*** **Hypomorphic variants with NF Kappa B activation**	300291	Fetal lethal or Dysglobulinemia. Recurrent infections, Osteopetrosis Abnormal teeth	Heterozygotes not affected	Yes, severe, with gradual elimination of mutant T cells	T cells	*AR*
Ectodermal dysplasia	Xq13.1 61,616,085	*EDA* Akin to tabby mouse missense, nonsense deletion, & splice junction variants	305100	Defective skin, hair, nails & teeth Variant interferes with rounding of cells by the cell membrane	Variable severity Skewing correlated with disease severity	No	WBC	*AR*
Epileptic encephalopathy early infantile, 1	Xp21.3 25,003,693	*ARX*	308350	Spasms without brain malformations	Milder than males	ND		
**Epileptic encephalopathy early infantile, 2**	**Xp22.13** **18,425,604**	**CDKL5** **deletions**	300672	Infantile seizures, global delay subtle dysmorphic features; most die early	Milder than males However, most heterozygotes affected…Males may die in utero? (32 deletions in females vs. 3 in males [Decipher])	No skewing	WBC	*AR*
**Epileptic encephalopathy early infantile, 2**	**Xp22.13** **18,425,604**	***CDKL5*** **missense**	300672	Some overlap with Rett; profound retardation and EEG abnormalities	Females less severe More apt to have hand stereotypies than males			
Epileptic encephalopathy, early infantile, 8	Xq11.1 63,634,966	Collybistin *ARHGEF9*	300429		Heterozygotes not affected unless skewed X-inactivation Manifesting females all have chromosome Translocation or deletion; 2 females with autism and intragenic deletions and no skewing	No, but manifesting requires complete skewing (mutant gene active) Speculation: skewing in brain but not in blood	WBC	*AR*
Epileptic encephalopathy, early infantile, 9	Xq22 100,291,643	*PCDH19*	300088	Not affected Mosaic males are affected	ID, Infantile seizures Autism	ND However, cellular interference thought to play a role		
Epileptic encephalopathy, early infantile, 22	Xq11.23 48,903,182	*SLC35A2*	300896	See *CDG2M*				
Fabry disease	Xq22.1 101,397,790	*GLA*	301500	Progressive heart & kidney disease	Attenuated Females express because not a high uptake enzyme	No	Skin fibroblast clones	*GLA*
Fanconi anemia	Xp22.31 14,690,862	*FANCB*	300514	Bone marrow failure, predisposed to cancer	No affected females	Yes, extreme	WBC	*AR*
**Focal dermal hypoplasia**	**Xp11.23** **48,508,991**	***PORCN*** **Loss of function**	305600	Fetal lethal Mosaic males survive, and have abnormalities, like females (10% of affected)	ID; skin atrophy & pigmentation; multiple papillomas; abnormal digits, striated bones; lobster claw	Microdeletions associated with severe skewing Point variants not skewed	WBC	*AR*
**Focal dermal hypoplasia**	**Xp11,23** **48,508,991**	***PORCN*** **(missense)**	305600	2 nonmosaic survivors; Missense variant inherited from mother	Random XI, but asymptomatic	No	WBC	*AR*
Fragile X syndrome	Xq27.3 147,911,918	*FMR1*	300624	XLID; congenital anomalies	Variable, milder	Yes, slight (full mutation)	WBC, skin fibroblasts	*FMR1* methylation
**Glycogen storage disease 1Xa1 GSD9A1**	**Xp22.13** **18,892,297**	***PHKA2*** **Complete loss of function**	306000	No PHK activity in liver & RBCs Yet mildest form of glycogen storage disease	Not usually affected	No	Skin fibroblasts	*PHK* activity
**Glycogen storage disease 1Xa2 GSD9A1**	**Xp22.13** **18,892,297**	***PHKA2*** **Missense enabling partial function**	306000	No PHK activity in liver; even milder than above	Heterozygotes not affected	ND		
Hemolytic anemia	Xq28 154,531,389	*G6PD*	305900	Chronic anemia	High dosages of primaquine; Rx for malaria cause hemolysis, if enough cells are mutant	Yes, slight with age	RBC, WBC	*G6PD*
Hunter syndrome (MPS2)	Xq28 149,505,353	*IDS*	309900	Mucopolysaccharidosis	Rarely affected unless skewed	No	Skin fibroblast clones	*IDS*
**Hydrocephalus, X-linked (due to aquaductal stenosis) allelic with MASA**	**Xq28** **153,861,513**	***L1CAM***	307000	Only males affected ID, spastic paraplegia Some have clasped thumb	Heterozygotes not affected	ND		
**MASA syndrome** **SPG1**	**Xq28** **153,861,513**	***L1CAM*** **same variants**	303350	Spastic paraplegia, aphasia, ID, abducted thumb, but no congenital hydrocephalus Family can segregate MASA or hydrocephalus phenotypes	Mild ID, abducted thumbs	ND		
Hypophosphatemic rickets	Xp22.1 22,032,324	*PHEX*	307800	Short stature; rickets; bone deformities	Heterozygotes variably affected	ND		
Ichthyosis	Xp22.31 7,147,289	*STS*	308100	Extensive body ichthyosis, corneal opacities	Late corneal opacities Expressed from XI	No (point variant)	Skin fibroblast clones Escape gene; 1/3 activity of XA	*STS* & *G6PD*
**Immunodeficiency 33**	**Xq28** **154,542,239**	***NEMO*** **variants disrupt leucine zipper**	300636	Infections limited to mycobacteria	Heterozygotes not affected	ND		
**Incontinentia pigmenti IP2** **Allelic with immunodeficiency 33**	**Xq28** **154,542,239**	***NEMO*** ***IKBKG*** **variants** **Usually deletions eliminating NF Kappa B activation**	308300	Fetal lethal usually; milder (hypomorphic) *IKBKG* variants lead to osteopetrosis in males only	Cell death causes rash along Blaschko lines Abnormal hair and teeth Females with hypomorphic IKBKG variants do not have osteopetrosis	Yes, severe skewing toward wild type, even with milder variants that permit male survival	Blood, WBC, Skin fibroblast clones	*HPRT* & G6PD
Immunodysregulation, polyendocrinopathy, and enteropathy (IPEX)	Xp11.23 49,250,435	*FOXP3* (*Scurfin*) *Scurfy* in mice	304790	Immunological disorder; diabetes mellitus, dermatitis and enteropathy, onset in infancy; death by 2 years unless treated by immunosuppression and blood cell transplantation; absence of islets of Langerhans; presents as severe diarrhea	Heterozygotes not affected	ND		
**Kabuki syndrome 2**	**Xp11.3** **44,873,174**	***KDM6A*** **or** ***UTX*** **(mediates removal of trimethylation of histone H3, at HOX promoters**, **demethylates H3K27; methylates H3K4)**	300867	ID Dwarfism, Kabuki facies, skeletal abnormalities; UTY protects	Like males Some say females less severe than males—perhaps due to skewing disfavoring deletions Escape may not protect females more than males as males also have an allele (UTY) on their Y chromosome	If deletion, then skewed; if variant, not skewed Escapes XI	WBC	*AR*
**Keipert syndrome** **Allelic with Simpson–Golabi–Behmel syndrome**	**Xq26.2** **133,300,102**	***GPC4*** **Missense variants** **Duplications cause Simpson–Golabi–Behmel syndrome**	301026	Craniofacial dysmorphisms; foot and hand abnormalities; mild intellectual disability	Carrier females are clinically unaffected, because all are >90% skewed toward wild type	Yes, severe in blood	WBC	*AR*
**Kelly–Seegmiller syndrome** **(gout, X-linked)** **Allelic with Lesch–Nyhan syndrome**	**Xq26.2** **134,460,164**	***HPRT*** **308000** **(partial, <95% deficiency)**	300323	Uric acid stones leading to gouty arthritis	Heterozygotes not affected	No	WBC	
**Lesch–Nyhan syndrome** **Allelic with X-linked gout**	**Xq26.2** **134,460,164**	***HPRT*** **308000** **(>98% deficiency)**	300322	ID, spastic cerebral palsy, uric acid stones, self-destructive biting	Heterozygotes not affected	Yes, severe (blood) No in skin because of gap junctions	RBC, WBC, skin fibroblast clones	*HPRT, G6PD*
Lissencephaly & agenesis of the corpus callosum	Xq22.3-q23 111,293,778	*DCX*	300067	ID, brain malformation due to neural migration defect seizures	Mild epilepsy (subcortical band heterotopia) or normal	No	Blood	*AR*
Lowe syndrome	Xq26.1 129,540,258	*OCRL1*	309000	ID; cataracts; rickets; aminoaciduria	Heterozygotes not affected	(100%) in one manifesting heterozygote unrelated to variant	WBC	*AR*
Severe systemic lupus erythematosis	Xp22.2 12,867,071	*TLR7*	300365	Rarely affected, except if XXY	Females 9 times frequency of males	ND Escape from inactivation in all females; B lymphocytes, monocytes and plasmacytoid dendritic cells		
Lymphoproliferative syndrome 2 XLP2	Xq25 123,859,811	*XIAP* inhibitor of apoptosis *BIRC4*	300079	Pancytopenia, splenomegaly, pancolitis	Usually heterozygotes not affected Occasionally female affected due to skewing toward mutant cells	Yes, in hematopoietic cells	WBC	*AR*
Lymphoproliferative syndrome *XLP1* (Duncan disease)	Xq25 124,346,281	*SH2D1A*	308240 300490	Severe immunodeficiency especially after EB virus infection; severe or fatal mononucleosis; acquired hypogammaglobulinemia; hemophagocytic lymphohistiocytosis (HLH), and/or malignant lymphoma	No reported affected females (probably because both skewing plus exposure to EB virus needed)	Carrier female had complete skewing toward wild type in NK cells but not in T or B cells	WBC	*AR*
**Mediator complex subunit 12** **MED12** **Allelic with Lujan–Fryns syndrome (309520); Ohdo syndrome (300895); Opitz–Kaveggia syndrome (305450)**	**Xq13.1** **71,118,595**	***MED12*** **or** ***HOPA*** **Transcriptional activator & repressor** **Different variants cause different syndromes** **Missense variant**	300188	ID plus tall stature (all hemizygous missense variants; different missense variants in same gene) ID plus macrocephaly; hypotonia; absence of corpus callosum	Infrequent & milder	See below		
**Lujan–Fryns Syndrome**	**Xq13.1** **71,118,595**	***MED12*** **or** ***HOPA*** **point variant in exon 22**	309520	Marfanoid habitus; long, narrow face; moderate ID;	Heterozygotes not affected	ND		
**Ohdo syndrome**	**Xq13.1** **71,118,595**	***MED12*** **or** ***HOPA*** **Missense variants**	300895	Blepharophimosis; ptosis; cryptorchidism; ID	Heterozygotes not affected	ND		
**Opitz–Kaveggia syndrome**	**Xq13.1** **71,118,595**	***MED12*** **or** ***HOPA*** **Missense variants** **C-T transition in exon 21**	305450	ID; macrocephaly; hypotonia & imperforate anus; partial or complete absence of the corpus callosum; often cryptorchidism	Much milder; hypertelorism; normal IQ	Variable; Four of 18 heterozygotes showed significant skewing but in different directions	WBC	*AR* & *FMR1*
**Melnick–Needles syndrome** **See OPD1**	**Xq28** **154,348,530**	***FLNA***	309350	1 of 4 otopalatodigital syndromes caused by variants in *FLNA*; most are severe; prenatal mortality or perinatal death Severe congenital abnormalities	Most affected have much milder phenotype; mild deformity of bones	Yes, likely skewed toward the normal allele because of cell selection	WBC	*AR*
**Menkes syndrome** **Allelic with occipital horn syndrome & spinal muscular atrophy**	**Xq21.1** **77,910,655**	***ATP7A*** **Truncation**	309400	Copper deficiency	Heterozygotes not affected	Yes, severe Caveat: 15-kb deletion	WBC, lymphoblasts, skin fibroblasts	*AR*
Methylmalonic acidemia Also referred to as XLMR 45	Xq28 153,947,555	*HCFC1* Host cell factor C1 300019 Missense	309541	Nonsyndromic ID	Normal IQ Highly skewed XI	Yes, severe	WBC Data not shown	
**Microophthalmia syndrome 2 (MCOPS2)** **Allelic with OFCD**	**Xp11,4** **40,051,245**	***BCOR*** **300485** **(See OFCD)** **Premature stop codons**	300166	Male lethal	Congenital cataract, microopthalmia Cardiac abnormalities Dental abnormalities	ND		
Microphthalmia, syndromic 7 (MCOPS7)	Xp22 11,111,331	*HCCS* microdeletions	300056	Male lethal Due to OXPHOS defect	Wide spectrum ranging from asymptomatic to corneal opacity; microphthalmia; linear skin defects; microcephaly; cardiac defects	Severe; complete or moderate (>80%) skewing Favoring normal allele (in blood cells)	WBC	*AR, MAOA, PGK FMR1*
Monoamine oxidase A deficiency (Brunner syndrome)	Xp11.3 43,654,906	*MAOA*	300615	Mild ID; aggressive impulsive behavior	Heterozygotes not affected	ND		
Muscular dystrophy, Duchenne	Xp21 31,119,218	*DMD*	310200	Muscular dystrophy Mild ID	Only when unrelated skew favors mutant cells	No	WBC	*AR*
Muscular dystrophy, Emery–Dreifuss	Xq28 154,379,235	*EMD*	310300	Muscular dystrophy; heart arrhythmias	No affected females	ND		
Myotubular myopathy	XQ28 150,673,142	*MTM1*	300415	Respiratory failure during 1st year; severe hypotonia	Asymptomatic, or mild weakness Some females skewed and symptomatic One female skewed 70:30 in muscle, and 55:45 in blood	NO or ND Histology (I think) looking for ragged fibers	55:45 method not presented	
**Neurodegeneration with brain** **iron accumulation (NBIA5)**	**Xp11.23** **49,074,432**	***WDR45*** **(all de novo)** **Most truncating or partial deletion**	**300894**	Fetal lethal, Mosaics survive to be affected	Static encephalopathy of childhood with neurodegeneration in adulthood; parkinsonism; dystonia; dementia	Only rare, severely skewed toward wild type females survive to manifest	WBC	*AR*
	**Xp11.23** **49,074,432**	***WDR45*** ***BPAN*** **variant**	**300894**	Occasional missense variant or germline mosaicism, but most males die in utero	Infantile spasms; developmental delay; ID; absent to limited language; Parkinson disease and dystonia develop with age	Skewed X-inactivation 2:98 in 11-year-old female Several older females with normal allele preferentially inactivated	WBC	*AR?*
Neurite extension & migration Factor	Xq13.3 74,732,855	*NEXMIF* formerly *KIA2022* See *XLID98*	300524	XLID	Less severe	No 36–64%	WBC	Methylation?
Night blindness (congenital) type 1A *CSNB1A*	Xp11.4 41,447,459	*NYX* (nyctalopia)	310500	Myopia and night blindness; rod function absent	14 daughters of 9 affected males were not affected However, some heterozygotes are and may reflect skewing—or are homozygous for the variant	ND		
Night blindness (congenital stationary incomplete) type 2A	Xp11.23 49,205,062	*CACNA1F*	300071	Nonprogressive retinal disorder with myopia and nystagmus No deterioration	6 obligate carriers had no symptoms	ND		
Nystagmus 1 (congenital)	Xq26,2 132,074,925	*FRMD7*	310700	Infantile, periodic, alternating	53% of carriers are affected Some have skewed X-inactivation	Findings not interpretable Perhaps wrong tissue analyzed	Actual data not shown One normal skewed; other normals at 50–60% Four affected skewed; two nonskewed	*AR*
Ogden syndrome Some variants cause microphthalmia syndrome (309800)	Xq28 153,929,224	*NAA10*	300855	Delayed psychomotor development; dysmorphic facial features; scoliosis, and cardiac dysfunction with long QT syndrome	1 severe female (with loss of function variant; another mild ID Wide spectrum depending on nature of variant; females with same variant as males are usually milder	4/4 nonaffected Heterozygotes have 90-100% skewing	WBC	*AR*
**Oculofaciocardiodental syndrome** **Microphthalmia syndrome 2**	**Xp11.4** **40,051,245**	***BCOR*** **null**	300166	Death in utero	OFCD Early onset cataracts Radiculomegaly of canine teeth Cardiac septal defects Facial dysmorphism	Some skewing toward wild type		
**X-linked BCOR-related syndrome** **Lenz microphthalmia**	**Xp11.4** **40,051,245**	***BCOR*** **variant** **C to T transition**	300485	Microphthalmia syndrome Severe microphthalmia Some variants have no eye abnormalities, but MR	Heterozygotes not affected	100% skewing		
**Orofaciodigital syndrome (OFD1)** **Allelic with Simson–Galabia–Beheld syndrome 2 & Joubert syndrome**	**Xp22.2** **13,734,712**	***OFD1*** **(*CXORF5*)**	**311200**	Fetal lethal A ciliopathy	Malformations of face & digits; polycystic kidneys	No human gene escapes XI In mice no escape and neonatal mouse females die of polycystic kidneys	WBC	*AR*
		**4**-**bp deletion in** ***OFD1*** **causing frameshift**				Mother (not affected) has random XI but affected daughter is skewed toward mutant allele		
**Joubert syndrome 10** **Allelic with Simpson–Golabi–Behmel, and OFD1**	**XP22.2** **13,734,712**	***OFD1*** **Small deletions**	300804	Ciliary dysfunction Hypotonia Cerebellar ataxia	Heterozygotes not affected	ND		
**Simpson–Golabi–Behmel syndrome type 2** **Allelic with OFD1**	**Xp22.1** **13,734,712**	***OFD1*** **4**-**bp deletion**	300209	One family of 9 males; most die early but one had severe ID, facial dysmorphisms, obesity, & repeated respiratory infections; respiratory cilia disorganized and uncoordinated	Heterozygotes not affected	ND		
**Oropalatodigital syndrome 1**	**Xq28** **154,348,530**	***FLNA*** **Mildest of spectrum**	311300	Cleft palate, conductive hearing loss; mild skeletal abnormalities & renal failure	Some mild symptoms	No, but skewing causes manifestation	WBC	*AR*
**Oropalatodigital syndrome 2**	**Xq28** **154,348,530**	***FLNA*** **Gain of function variants**	304120	Disabling skeletal anomalies, variable brain, cardiac, intestinal anomalies	Milder symptoms, some facial dysmorphism			
**Melnick–Needles syndrome**	**Xq28** **154,348,530**	***FLNA*** **Most severe**	309350	Fetal lethal	Skeletal dysplasia	Yes? Skewing in blood toward normal in 3 affected heterozygotes, but FLNA interacts with AR so assay may not be valid	WBC	
**Frontometaphyseal dysplasia**	**Xq28** **154,348,530**	***FLNA***	305620	Frontal bone overgrowth, scoliosis, facial dysmorphism; increased bone density; occasional renal abnormality	Normal, or mild hyperosteosis	ND		
Ornithine transcarbamylase deficiency	Xp11.4 38,352,527	*OTC*	311250	Urea cycle disorder; males die in infancy of severe disease unless treated	85% are symptomatic with hyperammonemia 20–30% activity not enough	No, but skewing in liver, not WBCs, corresponds with severity	WBC & Liver	
PDC deficiency	Xp22.12 19,343,892	*PDHA1* pyruvate dehydrogenase complex, E1-ɑ polypeptide 1	312170	a) Neonatal lactic acidosis; encephalopathy; brain malformations; early death b) Infantile or childhood-onset Leigh syndrome c) Milder relapsing disorder of ataxia dystonia and peripheral neuropathy	Dysmorphic features; microcephaly; moderate or severe psychomotor delay; spastic di/quadriplegia & epilepsy (cortical atrophy, cyst & corpus callosum agenesis); all heterozygous; more severe variant than in males Missense variants are milder	No, but skewing determines severity of phenotype	WBC	*AR*
Pelizaeus–Merzbacher	Xq22.2 103,776,505	*PLP1* point variant Often duplications of Xq22.2	312080	Myelin leukodystrophy Spastic diplegia	No symptoms or milder; Rare female affected due to skewing toward mutant cells Some mild affected have no skewing because variant not severe enough to skew	During CNS development, oligodendrocytes with severe *PLP1* mutant alleles are negatively selected (apoptosis) in favor of wild type cells, with cell type specific skewed XI	WBC, lymphoblasts	*AR*
PIH1D3	Xq22.3 107,206,610	*PIH1D3* formerly *Cxorf41* Expressed primarily in testes	300933	Primary ciliary dyskinesia; nonsyndromic ODA; respiratory infections; chronic otitis; infertility with mutant sperm	Heterozygotes not affected Normal fertility	ND		
**Phosphoribosyl pyrophosphate** **synthetase 1 spectrum**	**Xq22.3** **107,628,423**	***PRPS1*** **Gain of function**	300661	Hyperuricemia; gout; deafness and neurological misfunction	His mother had gout, uric acid stones and hearing loss	ND		
**Charcot–Marie–Tooth**	**Xq22.3** **107,628,423**	***PRPS1*** **Reduced expression**	311070	Peripheral neuropathy; deafness; visual impairment; no increased uric acid	Not affected or milder	Variable skewing consistent with phenotype	WBC	*AR*
**Arts syndrome**	**Xq22.3** **107,628,423**	***PRPS1*** **Reduced expression**	301835	ID; hypotonia; ataxia; hearing impairment and optic atrophy	Milder but continuous spectrum depending on extent of skewing	Variable skewing consistent with phenotype	WBC	*AR*
**DFNX1**	**Xq22.3** **107,628,423**	***PRPS1*** **Missense variant** **40–70% reduced activity**	304500	Deafness	Spectrum of phenotypes Usually milder	ND		
Protoporphyria (X-linked erythropoietic)	Xp11.21 55,009,054	*ALAS2* Increased activity gain of function	300752	RBC porphyria	11 females vary in phenotype with skewing of XI correlated with degree of severity Example of no X-linked dominant disease	No selection, but skewing influences phenotype	Blood DNA	*AR* & *ZMYM3*
Raynaud–Claes syndrome	Xp22.2 10,156,944	*CLCN4* Chloride/hydrogen ion exchanger truncated and missense frameshift	300114	Severe ID & epilepsy Impaired language	Milder	Not shown; No obvious selection	WBC	?? Poor study
Renpenning syndrome	Xp11.23 48,897,861	*PQBP1* Polyglut binding protein 1	300463	MR; short stature, microcephaly	Unaffected, but skewed XI	Highly skewed	WBC	*AR, FMR1*
**Retinitis pigmentosis 3**	**Xp11.4** **38,269,162** **Loss of function; hot spot in exon 15**	***RPGR***	300029	Inherited choroidal retinal degeneration; destroys rod photoreceptors; become blind	All heterozygotes have tapetal-like retinal reflex; some heterozygotes are affected—usually milder, but variable; rarely legally blind	ND		
**X-linked cone–rod dystrophy**	**Xp11.4** **38,269,162** **variants in alternate exon 15**	***RPGR***	304020	Progressive loss of vision	Not affected, but detectable by visual testing	ND		
**Syndromic retinitis pigmentosa**	**Xp11.4** **38,269,162**	***RPGR***	300455	RP3 with sinorespiratory infections, with or without deafness	Often affected, but milder	ND		
**Rett syndrome**	**Xq28** **154,021,572**	***MECP2*** **point variant**	312750 300005	Fetal lethal; mosaic males have Rett syndrome; some males without obvious mosaicism survive; severity dependent on mosaicism & variant	MR; arrested development; impaired speech; handwringing	No, unless large deletion; Blood DNA does not always reflect brain DNA	WBC	*AR*
**Lubs XLID** **Allelic with Rett syndrome**	**Xq28** **154,021,572**	***MECP2*** **duplication**	300260	Affected males have Rett phenotype; moderate to severe ID; seizures	Unaffected because of extreme skewing	Extreme skewing	WBC	*AR*
**Sick sinus syndrome**	**Xq28** **154,021,572**	***MECP2*** **deletion** **(0.6 deletion effecting TDR)**	Not yet given	Autonomic NS affected in two brothers	Milder symptoms	Extreme skewing in unaffected mother	WBC	*AR*
Simpson–Golabi–Behmel syndrome type 1	Xq26.2 133,535,744	*GPC3* Duplication of *GPC3* & *GPC4* produce some affected females, but skewing toward mutant needed	312870	Congenital malformations & overgrowth ID, macrocephaly, cleft palate	Rare; usually unaffected or much milder Rarely affected; Female with a small Xq26 microduplication is affected; she has random XI	No; skewing toward mutant produces symptoms Normal females not skewed	?	
**SCIDX1** **Severe combined immunodeficiency**	**Xq13.1** **71,107,403**	***IL2RG***	300400	B- & T-cell immunodeficiency	Heterozygotes not affected	Yes, extreme	T & B cells	Cell count
**CIDX1**	**Xq13.1** **71,107,403**	***IL2RG***	312863	Milder allele of SCIDS	Heterozygotes not affected	Yes	Predominantly affects T cells,	*AR*
Tonne–Kalscheurer syndrome	Xq13.2 74,582,975	*RLIM* *RFN12*	300379	Global developmental delay; ID; subtle facial dysmorphism; multiple congenital anomalies autism; severe feeding problems	Heterozygotes not affected unless severe loss of function	All 4 females with mild skeletal anomalies were extremely skewed (not shown), and normal females also skewed Direction of skewing not shown	WBC	*AR*
**Thrombocytopenia** **Allelic with Wiskott–Aldrich**	**Xp11.23** **48,683,752**	***WAS*** **hypomorphic alleles**	313900	Decreased number of platelets; bleeding tendency	Rare, presumably due to chance skewing	Reported as random but results not interpretable	WBC	*AR*
**Wiskott–Aldrich syndrome**	**Xp11.23** **48,683,752**	***WAS*** **severe loss of function**	301000	Deficiency of B & T cells, leukocytes & thrombocytes	No affected females	Yes, severe	T, B cells & granulocytes	*AR*
**XLID 1 & 18, 78** **Allelic with** **1Q motif and SEC7 Domain** **18M,19F XLID**	**Xp11.2** **53,225,783**	***IQSEC2*** **Nonsense & duplications**	300522	ID nonsyndromic	Some have learning disabilities	ND, escapes XI		
**IQ Motif and SEC7 Domain** **18M,19F** **XLID** **allelic with XLIDR 1, 18 and 78**	**Xp11.22** **53,225,783**	***IQSEC2*** **(Shapes dendrite morphology) Truncating variants**	300522	ID nonsyndromic; 95% have epilepsy variants reduce axon length in mice; de novo or maternal	Milder ID 70% epilepsy without seizures—all de novo 4/5 affected had random XI 1 skewed 2 nonaffected carriers had random inactivation	No, 1 severely affected female favoring mutant allele Human gene escapes XI, but not mouse gene	Blood DNA	*AR*
XLID 12 Also called THOC2 XLID	Xq25 123,600,562	*THOC2*	300395	Mild–moderate ID Speech delay Neurological developmental defect	Heterozygotes not affected			
XLID 15 Cullin Ring Cabezas type	Xq24 120,524,588	*CUL4B*	300354	ID (IQ 29–54), short stature macrocephaly, hypogonadism (small testes), tremor, abnormal gait	Rarely mild tremor, tics Mice die due to PXI Obligate heterozygotes have large hands some learning disability	Yes, severe	WBC	
**XLID Turner**	**Xp11.22** **53,532,095**	***HUEW1*** **E3 ubiquitin ligase** 300697 **Microduplications**	309590	Moderate to profound ID, global delay with macrocephaly nonsyndromic	Heterozygotes not affected	Yes & no: severe favoring wild type in some females with microduplications	WBC	*AR*
	**Xp11.22** **53,532,095**	***HUEW1*** **Missense variants** **codes E3 ubiquitin ligase**	309590	Moderate to profound XLID; short stature; speech pathology; small hands & feet	Heterozygotes not affected			
	**Xp11.22** **53,532,095**	***HUEW1*** **De novo loss of function**	309590	No affected males seen	Females with dysmorphic XLID; craniostenosis; Chiari malformation	Severe skewing favoring mutant in affected females		
XLID Claes–Jensen	Xp11.22 53,176,276	*KDM5C/JARIDC* 314690 Also, *SMCX*	300534	Short stature; microcephaly; abnormal facies; developmental disability	Milder	Yes, severe favoring wild type in 4 unaffected heterozygotes	WBC	*AR*
XLID Houge type (MRXSHG)	Xp22.2	*CNKSR2* Deletions; frameshifts; truncating variants	301008	Delayed psychomotor development; poor verbal skills; microcephaly; focal seizures	Mild learning disability or nonaffected Speculation that skewed XI prevents severe effects in brain, but not shown, nor is it needed	Not shown, but 2 manifesting heterozygotes had 56:43 and 20:80 XI ratios *CNKSR2* is only expressed in brain tissue	WBC	
XLID Siderius	Xp11.22 53,936,679	*PHF8* Microdeletion (470 kb)	300263	ID; abnormal facies; cleft palate/lip	Heterozygotes not affected	Yes, severe favoring wild type in unaffected mother,	WBC	*AR*
XLID 90	Xq13.1 70,444,834	*DLG3* Truncating	300850	Moderate to severe nonsyndromic ID	Heterozygotes are usually not affected	Majority of heterozygotes have random XI Skewing influences phenotype		
**XLID 98**	**Xq13.3** **74,732,855**	***NEXMIF*** **(KIAA2022)** **Hypomorphic loss of function**	300524	Severe ID; poor speech postnatal growth retardation Strabismus	Usually not affected unless truncating variants (see below)	Normals not studied (one inversion showed skewing) 1 affected female not skewed (73:27)	WBC	*AR*
	**Xq13.3** **74,732,855**	***NEXMIF*** **(KIAA2022)** **Heterozygous truncating variants—all loss of function**	300524	NA	14 females with intractable epilepsy, milder ID	6/7 had random XI 1/7 (the most severe) had completely skewed XI favoring mutant cells	WBC	? Data not shown
**XLID 99 Male restricted** **(MRXS99F)**	**Xp11.4** **41,085,419**	***USP9X*** **Nontruncating missense variants, no effect on catalytic activity**	300919	ID, hypotonia, aggression, thin corpus callosum; loss of hippocampal-dependent learning & memory	Female carriers identified and none affected	ND *USP9X* escapes XI in some tissues, not in others		
**XLID 99 Female restricted**	**Xp11.4** **41,085,419**	***USP9X*** **30072** **Point variants leading to truncation** **De novo loss of function variants**	300968	Male lethal Truncating variants are lethal in males	MR; short; choanal atresia; heart defects; polydactyly; hearing loss; skin pigmentation (Blaschko)	Affected females may be skewed >90% but test does not show direction; *USP9X* escapes XI in some tissues	WBC	*AR*
**XLID 102X**	**Xp11.4** **41,333,307**	***DDX3X*** **complete loss of function 300160**	300958	LOF lethal (Decipher shows loss of males)	LOF causes MR, spasticity, ASD Heterozygotes with hypomorphic variant are not affected	No *DDX3X* escapes XI Homolog on Y		
	**Xp11.4** **41,333,307**	***DDX3X*** **Hypomorphic variants**		Occasional males have nonsyndromic ID	No disease	ND		
**XLID 106**	**Xq13.1** **71,533,103**	***OGT*** **Missense**	300997	IQ 49–61; facial dysmorphisms Mild spastic diplegia plus other congenital abnormalities Embryonic lethal (mice)	Females normal but highly skewed X-inactivation	No reference provided In mice, if maternal allele mutant then embryonic lethal		
**XLID 106**	**Xq13.1** **71,533,103**	***OGT*** **Missense**	300997	Usually only males	Twin sisters XLID plus some facial dimorphisms	Each had 93:07 Direction of skewing not known	WBC	*AR*
XLID 107	Xq24 119,538,148	*Cxorf56* 301012 Frameshift variant	301013	5 males: Moderate ID, Long narrow face	Milder 1 female with no skewing (57%) but nonaffected carriers were skewed	Normal Significantly skewed (76–93%)	WBC	*AR*
**XLID Wilson–Turner** **See Cornelia de Lange 5**	**Xq13.2** **72,329,515**	***HDAC8*** **Point variants**	300269	ID, microcephaly craniofacial deformities	Milder	Yes, extreme	WBC	*AR*

The X-linked diseases in this table are disorders with available information about heterozygous females. They are listed with their disease name, their X map locus (the location of the gene on the X chromosome, including its 5’ start site, from OMIM). These data are followed by the symbol for the variant gene, the protein that is deficient and the nature of the variant if known. In each case, although the phenotype is variable, the most common one is described. Cell selection favors cells expressing the normal allele, unless otherwise indicated. In all cases chance skewing, that is skewing unrelated to the variant, influences the phenotype. Allelic disorders are indicated in bold.

*AR* androgen receptor, *ASD* autism spectrum disorder, *ID* intellectual deficiency, *LOF* loss of function, *SCIDS* severe combined immunodeficiency syndrome, *XLID* X-linked intellectual deficiency, *WBC* white blood cells.

## SEX DIFFERENCES ARE DUE TO X-INACTIVATION

The sex differences in the effect of X-linked pathologic variants is due to our method of X chromosome dosage compensation, called X-inactivation;^9^ humans and most placental mammals compensate for the sex difference in number of X chromosomes (that is, XX females versus XY males) by transcribing only one of the two female X chromosomes. X-inactivation silences all X chromosomes but one; therefore, both males and females have a single active X.^10,11^

For 46 XY males, that X is the only one they have; it always comes from their mother, as fathers contribute their Y chromosome. However, because X chromosomes are silenced in a random fashion, females usually have two kinds of cells in every tissue; those with their maternal X active and those with an active paternal X. Females are protected to a large extent because their two X chromosomes most often differ in genetic content.

**Fig. 1:**
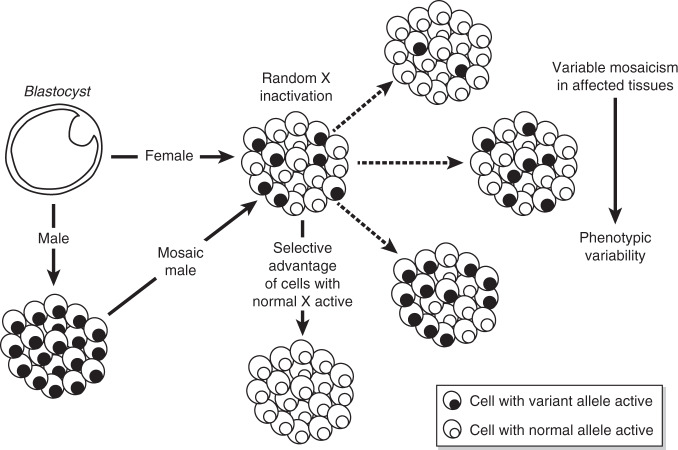
Comparing the effect of a single pathologic variant in females and males. Adapted from Fig. 1 of Franco B, Ballabio A. Curr Opin Genet Dev. 2006;16:254–259, with permission of authors. In females: On average, the ratio of the two cell types (expressing normal or variant alleles) is approximately 50:50. However, the ratio may differ because of chance, or a selective disadvantage for cells expressing the variant. Extreme divergence from the 50:50 ratio, known as skewing of XCI, may differ from tissue to tissue, and among individuals influencing the severity of the phenotype. Cell selection usually takes place only in cells which express the variant and which do not receive the essential gene products from the normal cells that make them. In males: an X-linked variant is expressed in every cell. The exceptions are males with somatic mosaicism: like females, mosaic males will express both variant and normal allele, and the phenotype depends on the admixture of variant and normal cells.

Sex differences in diseases due to deleterious variants encoded by the X chromosome originate from the sex difference in the expression of the variant allele; if present in male tissues, it is expressed in every cell, but if present in female tissues, the variant is usually expressed in only half the cells (Fig. 1).

## FEMALES ARE MOSAICS

A woman is less susceptible to the pathogenic variants in genes on her active X chromosome because the variant is not expressed in all her cells.^12^

## FEMALES CAN AMELIORATE THE EFFECTS OF PATHOGENIC VARIANTS

Most women do not manifest X-linked disorders because (1) they are not homozygous for the pathogenic variant, and (2) their variant cells (those expressing the deleterious allele) receive sufficient gene product to carry out the essential metabolic function from the cells that transcribe the normal allele.

The crucial protein is provided in one of two ways. Either the cells that can make it transfer it to the deficient cells, or, if this cell-to-cell transport does not take place, the lack of functional protein may cause the deficient cells to divide less efficiently, and so they are eventually overgrown by the cells that make the normal protein. Yet, in various tissues, cells differ in their capacity to transfer gene products and so there may be differences within body tissues in the ability of the normal cell to share, or the abnormal cell to survive^12^ (Fig. 1, Table 1).

## FEMALES ALMOST ALWAYS HAVE LESS SEVERE MANIFESTATIONS OF X-LINKED DISEASES

For most X-linked deleterious variants, the manifestations are less severe in females than males. The mix of normal and abnormal cells moderates females' disease. Yet, there are disorders in which the variant is so lethal that most males with severe deficiency of the gene product die in utero. Because the only survivors are females or mosaic males, who also have a mix of variant and normal cells, they are the ones with nonlethal manifestation of the disorder. X-linked diseases, such as incontinentia pigmenti, or orofacial digital syndrome type 1, occur only in females or mosaic males.^13^

## SOME FEMALES ARE MANIFESTING HETEROZYGOTES

Many factors determine if a heterozygote manifests her mosaic variant. Our knowledge of the effect of second variants of genes in related pathways on the ultimate phenotype is meager, but suggests that we need to be aware of the potential effect of other relevant genes on any variant. Although Table 1 includes only one known instance of digenic X variants (Dent disease 2), one expects there will be more. The effect of digenic variants leading to Dent disease are ameliorated in the heterozygote, who carries both variants on the same X chromosome, as these abnormal cells are strongly disfavored (Table 1).^8^ In this case, heterozygotes are much less severely affected than hemizygous males because of the strong cell selection against cells expressing the two variants simultaneously.

## EFFECT OF SEX AND X-INACTIVATION ON PHENOTYPE IN X-LINKED DISORDERS

As I learn about why some heterozygotes express their variants, yet others with the same variant do not, I more fully appreciate the nuances of disease processes and the intricacy of the interdependence between the type of cell, its response to abnormal gene products, and the nature of the disease variant. Table 1 shows the effect of pathogenic variants of X-linked genes in males and females. For almost all of the disorders listed, females have less severe clinical manifestations. Table 1 also documents the variables that determine if an X chromosomal variant is manifested or not, that is, if products are shared or if normal cells have a selective advantage. The severity of the phenotype is most often attributable to the nature of the variant. Does it completely eliminate the gene’s function, or does it allow some gene product to be produced? Variants that disable an essential protein completely are more often seen in females, as males whose only gene is nonfunctional are lost in utero, unless other genes can substitute for it. On the other hand, variants with some residual function may affect only males. In both cases, the female phenotype is less severe, as she has some normal cells that carry out the essential function.

## EFFECT OF SHARING GENE PRODUCT BETWEEN CELLS

Because it is largely unknown, Table 1 does not include the quantity of gene product that must be provided to a cell to replace that lost through a variant. In most cases 50% activity is more than enough, and for many genes, even smaller amounts of product will suffice. This readily explains why many X-linked disorders never affect women. For example, less than 5% of the enzyme HPRT can alter the phenotype from severe hyperuricemia, seen in Lesch–Nyhan males, to gout. Lesch–Nyhan heterozygotes rarely express any features of the syndrome, including gout. In most of her tissues the product of the HPRT reaction, inosinic acid, is transferred from her cells that synthesize it, to those that cannot, through gap junctions, present in all cells of the body except blood cells.^14^

Cell sharing also occurs in women who are heterozygous for variants causig X-linked lysosomal diseases. Lysosomes contain many enzymes that break down proteins and lipids. Variants in the genes that encode these enzymes cause diseases by the accumulation of undegraded material within the lysosomes of affected individuals. Fortunately, the deficient cells of the heterozygote can take up the enzyme secreted by the normal cells through a process called endocytosis. Therefore, potential manifestations in carriers of variants of lysosomal enzymes encoded by the X chromosome are generally ameliorated by the transfer of these enzymes from the cells that can make them to those that cannot.^15^

## EFFECT OF CELL SELECTION IN MOSAIC FEMALES

Because they lack gap junctions, the leukocytes from Lesch–Nyhan heterozygotes do not receive the inosinic acid made by their normal counterparts. Fortunately, the lack of inosinic acid slows the rate of cell division; the normal cells (because the variant is on their inactive X) eventually replace the deficient ones. As a consequence, heterozygous mothers and sisters of Lesch–Nyhan males have only normal blood cells, and in other tissues, their mutant cells share the gene products provided by their normal cells.^16^ It takes about ten years for all their abnormal red cells to be replaced^17^ because in this case, the selective advantage of the normal allele is relatively small.

The rate of loss of deficient cells can be slow or fast depending on the degree to which the variant is disadvantageous. When selection is intense, that is, when it severely disfavors the cells that express the variant, heterozygous females benefit, as they rapidly lose all their mutant cells. Sometimes the loss of variant cells occurs so early that abnormal cells may never enter a tissue. In cases of immunodeficiencies, like Wiskott–Aldrich syndrome, the growth disadvantage is immediate; the mutant B cell precursors never leave the bone marrow (Table 1).^8^

Unfortunately, sometimes it is the variant cell that has the selective advantage. For reasons not yet understood, heterozygotes with the variant causing adrenoleukodystrophy slowly lose their population of wild type cells.^18^ Therefore, as they age, they usually manifest some symptoms of the disease. Adrenoleukodystrophy is the only known X-linked disease where the variant allele has a selective advantage. X-linked variants that slow the growth of the expressing cells usually protect females from manifesting them. If one examines the cells from these heterozygotes, one sees highly skewed patterns of X-inactivation in the expressing tissues: eventually, the X with the variant will always be silent (that is, always on the inactive X). Females carrying pathologic variants that produce X-linked mental retardation often have X-inactivation patterns that are highly skewed favoring expression of the wild type gene, protecting them from ill effects of their variant.^19^ In such women, the cells that express the variant gene are disfavored.

## MANIFESTING HETEROZYGOTES

With so many mechanisms available to protect females with an X-linked pathologic variant, one wonders why some heterozygotes manifest any symptoms of a disease. As mentioned previously, if the variant is so functionless that it is lethal to fetal or newborn males, then females with a single mutant allele are the only ones that can have the disease. As Table 1 indicates, the severity of the variant can determine if females express the variant or not.

Occasionally, females are the ones to manifest a disease because the variant allele interacts with the normal one, undergoing a process called cellular interference. The most well defined example of this is the craniofrontonasal syndrome, caused by a deficiency of Ephrin-B1 (*EFNB1*).^20^ Other members of the ephrin family of proteins can substitute for a deficiency of *EFNB1* in males with the deleterious variant, and as a result, they have minimal clinical symptoms. Heterozygotes, however, have a mixture of mutant and wild type cells; for reasons not yet understood, it seems that such mixtures do not permit ephrin substitutes, and consequently, females have a deficiency more severe than that in males, who can substitute one ephrin for another. It is heterozygosity, not the complete loss of function, that produces the severe disorder. It is the patchwork or mosaic loss of *EFNB1* that disturbs tissue boundary formation at the developing coronal suture. Several forms of infantile epilepsy also show similar cellular interference, but fortunately no other examples are known.

Yet, there are females who express an X-linked pathogenic variant, even though most carriers of pathogenic variants in similar genes do not. For example, females with Fabry disease, caused by lack of the lysosomal enzyme ɑ-galactosidase may have some of the clinical symptoms seen in affected males, whereas carriers of a variant in a gene encoding another lysosomal enzyme, iduronic sulfatase, rarely have any clinical symptoms associated with Hunter syndrome.^21^ Both enzymes are made in the lysosomes, and can be transported from the lysosomes of one cell to those of another cell. The two lysosomal disorders differ because iduronate sulfatase is taken up by cells better than the low uptake enzyme, ɑ-galactosidase.^22,23^ This difference in the ability of the lysosome to take up a product is responsible for normal Hunter heterozygotes and manifesting Fabry heterozygotes.

## OTHER DETERMINANTS OF MANIFESTING HETEROZYGOTES

One wonders why some heterozygotes express disorders that do not affect most of the others with the same variant. Some females manifest their X-linked variant because it is overexpressed; their variant is expressed in more than half their cells because of skewing in the proportions of normal and abnormal cells. Although random inactivation usually means that 34–68% of heterozygous cells are abnormal,^12^ a few heterozygotes have more than 90% variant cells. Such females manifest the disorder because their wild type allele is not expressed sufficiently. These manifesting heterozygotes are often reported in medical journals because they are affected with a disease that usually occurs only in males. In some cases, a chance chromosome translocation or deletion is responsible for the skew, as such abnormal X chromosomes often influence the direction of skewing. X/autosome translocation chromosomes are responsible for the rare females afflicted with Duchenne muscular dystrophy.^24^ Maternal isodisomy,^25^ early onset monozygotic twinning (known to promote skewing^26,27^), and other extreme skewing of X-inactivation^28^ have also been responsible for manifesting heterozygotes (Table 2).

**Table 2:** Factors influencing expression of X-linked variant in females.

Variant		X-inactivation				Outcome^a^	
Number of mutant alleles	Strength of variant	Random	Skewed To variant^b^	Skewed To wild type^c^	Escape allele^d^	Males	Females
Biallelic (rare)	NA	NA	NA	NA		Affected	Equally affected as male
	NA	NA	NA	NA	+	Affected	Equally affected or worse than male
Monoallelic	Mild to moderate	**+**				Affected	Less severe or no abnormalities
	Mild to moderate		**+**			Affected	Equally affected
	Mild to moderate				**+**	Affected	Less or more severe
	Severe	**+**				Fetal lethal	Express variant
	Severe			**+**		Fetal lethal	Less severe
	Severe		**+**			Fetal lethal	Severe or lethal
	Severe				**+**	Fetal lethal	Less or more severe
	X/A translocation			**+**		Affected	Less severe
	X/A translocation		**+**			Affected	More severe

^a^Male phenotype is given; female phenotype is given relative to male.

^b^Variant allele is on the active X.

^c^Normal allele is on the active X.

^d^Allele is also expressed to a small extent from the inactive X.

Although some skewing may be attributable to abnormalities of the *XIST* locus,^29^ such variants occur only rarely. If one *XIST* allele loses function, than that allele is always on the active X. Minor loss of function variants of *XIST* may lead to some skew,^29^ but few have been reported. Cell selection and random skewing are the most frequent causes of nonrandom X-inactivation. Random skewing frequently occurs because of events surrounding twinning, and confined placental mosaicism.^30^ Often skewing is due to the randomness of the inactivation process, which is stochastic and therefore due to chance. Ten percent of females are >2 standard deviations from the mean of 50%.^23^

Stochastic skewing that favors cells that inactivate the normal *WDR45* allele is responsible for neurodegeneration seen in the rare female infants who manifest the X-linked pathologic variant (*NBIA5*), because their brains accumulate iron. If this disorder is caused by a truncating variant, only mosaic males, or females who are highly skewed favoring the normal allele, manifest the disease, as all other males and females with the variant die in utero. Affected females with hypomorphic variants often skew favoring the mutant allele (Table 1).^8^

## DEGREE OF CELL SELECTION IS DETERMINED BY THE VARIANT

The X-linked form of Kabuki syndrome is caused either by point variants or deletions in *KDM6A*. Females with point variants often have Kabuki syndrome, whereas those with the larger deletions silence the X carrying the deletion; hence, they have less severe manifestations.^31^

Only a rare heterozygote with a variant in the *PLP1* gene has symptoms of the myelin leukodystrophy associated with Pelizaeus–Merzbacher syndrome, and she invariably shows chance skewing favoring the mutant cells.^32^ In addition, during the central nervous system (CNS) development of heterozygotes, the oligodendrocytes with severe *PLP1* disease alleles are negatively selected (apoptosis) in favor of wild type cells, resulting in skewed X-inactivation that is cell type specific.^8^

## ESCAPE GENES INFLUENCE PHENOTYPE

Another factor that influences the clinical manifestations of X-linked variants in heterozygotes is the partial expression of genes from the inactive X chromosome.^33^ In addition to those genes in the pseudoautosomal region that are expressed on both sex chromosomes, more than 100 genes on the human X chromosome are expressed not only from the active X, but also to some extent from the silent X.^34^ They are referred to as escape genes. In fact, such genes do not really escape silencing, as they function to some extent, usually producing 10–30% of the level of transcripts made by the homologous allele on the active X. Yet, this little extra expression of a gene does influence phenotype. For example, male fetuses with pathologic variants causing orofacial digital syndrome all die in utero, unless they have a second X chromosome (i.e., Klinefelter syndrome); human females survive birth and die in their forties, usually from renal failure. However, female mice with the same variant outlive males by only several weeks as they die as neonates due to their polycystic kidneys. The important species difference is that humans, but not mice, partially express the *OFD1* gene from their inactive X.^13^ This little extra gene activity is responsible for the difference in age of death of females of the two species. A little extra gene product from the inactive X can ameliorate the effects of an X-linked variant (see Table 2).

The extra product may not be available in every tissue, as there are tissue-specific differences in the expression of escape genes. In several females with a *USP9X* variant, pigment changes along the Blaschko lines and body asymmetry were observed, which is probably related to differential escape from X-inactivation between tissues^35^ and Table 1.

Yet, a little extra activity from the inactive X is not always helpful. It seems that expression of the Toll-like receptors on the inactive X is partly responsible for the impressive sex differences in some cases of autoimmune diseases like Lupus and Sjogren disease.^36^ Toll-like receptors, encoded by the X chromosome, are signaling pattern recognition receptors that are an integral part of innate immunity. The little extra activity may provide females with better protection against infectious agents, but it may make them more susceptible to autoimmunity. Such disorders are nine times as frequent in females than males and are also increased in Klinefelter XXY males, whereas as Turner females, even those taking estrogens, have the same risk as XY males. It is now thought that the excess manifestations of autoimmune disease in women are due to a complex of issues. Studies in mice show that increasing the expression of the X-linked Toll-like receptors in susceptible mice increases the expression of the autoimmune disorder.^36^ It has not yet been shown that affected females and Klinefelter males have greater expression of their Toll-like receptors than those women and XXY males who do not have the disorder.

Other relevant X-linked genes with potential to influence autoimmunity and that escape X-inactivation include *CXCR3*,^37^ *KDM6A*,^38^ and *CXorf21*, which has been shown to be more highly expressed in women and Klinefelter males than in normal men.^39^ In addition, the expression of *XIST* from the inactive X in lymphocytes differs significantly from that of other cells as the *XIST* RNA cloud is dispersed, leading to poorer Barr body formation.^40^ No doubt, it is the interaction of several factors that is responsible for the high incidence of autoimmunity associated with having two X chromosomes.

## DIFFERENT DISORDERS FROM THE SAME GENE

What is increasingly apparent is that variants in a single gene can lead to differently named disorders, because of the specific effect of the variant on production of the gene product. Before variants were identified, diseases were classified by their phenotypes and named by the physician who reported the disease, based on its symptoms. Now, we know that many different phenotypic disorders may be due to variations in the same gene; their effect in the heterozygote reflects the severity of the variant, which has influenced the naming of the disorder. Table 1 shows some of these allelic disorders (in bold). Yet, in most cases, no matter the severity of the disease in males, the heterozygous female is better off than the hemizygous male.

Examples of allelic disorders are the different variants in the IKBKG gene, which can cause incontinentia pigmenti (IP) if dysfunction is severe, ectodermal dysplasia if dysfunction is moderate, and immunodeficiency 33 if it is mild. When the pathologic variant is severe (like IP), females may be the only ones that manifest the disorder, as males die in utero. On the other hand, when it is moderate, or mild (like immunodeficiency 33), women may completely escape its pathologic effects (Table 1).^8^

Another set of allelic disorders is caused by variants in the *PRPS1* gene, responsible for syndromes of deafness (missense variant leading to reduced expression), gout (gain of function variant), and Charcot–Marie–Tooth disease (more severe reduction of expression), depending on the severity of the specific variant. Again, the heterozygous female always has a less severe phenotype than the hemizygous male. When the male is deaf, the female has high range hearing loss; when he has gout, she has no manifestations. However, when he has optic atrophy and neuropathies, she has much milder manifestations, or none at all (Table 1).^8^

There is a spectrum of allelic disorders caused by different variants in the filamin A gene; again females are always less affected than males with the same deleterious variants, and are the only ones affected with Melnick–Needles syndrome, because it is lethal in males (Table 1).^8^

There are also allelic disorders caused by different variations in the *MECP2* gene. Rett syndrome results when the gene has a deletion or substitution variant that decreases its function; the loss of function causes a disorder that is usually lethal to unborn males, so that generally only females survive to manifest the disease. However, when the same gene is duplicated (Lubs X-linked intellectual disability [XLID], Table 1), the increased function permits males to survive and manifest the disorder, whereas the gene duplication provides the normal allele a selective advantage, so that females escape all manifestations, as the duplicated gene is always on the inactive X (Table 1).^8^

Another impressive example of the effect of allelic disorders on sex differences in expression of the disease are variants in the *USP9X* gene (Table 1).^8,35^ As point variants leading to truncation are lethal for the male fetus, the manifestations are confined to females. On the other hand, less severe nontruncated variants in the same gene cause hippocampal related mental retardation, hypotonia, and aggression in males, whereas carrier females have no abnormalities.

## MANY X-LINKED GENES ARE ASSOCIATED WITH INTELLECTUAL DEFICIENCY

The proportion of X-linked variants causing intellectual deficits is striking. In addition to the many X-linked variants whose phenotypes include intellectual disability, there are many disorders that are *specifically* associated with X-linked intellectual deficiency, both syndromic and nonsyndromic; these are listed in OMIM as XLID followed by a number from 1 to 107. These disorders map from the telomere of the short arm to the last band on the long arm of the X. Although the role of the gene whose variant leads to the disorder is not always well defined, the genes responsible for X-linked intellectual disability are involved in many pathways. It seems that intelligence is the sum total of how all our genes are functioning. Malfunctions of genes in many pathways can interfere with intellectual capabilities. Extreme skewing of X-inactivation is frequently seen with XLID variants that permit male survival, and the variant is always found on the inactive X in females; consequently, they have normal intellectual function. Apparently, such disease-producing variants are toxic for expressing cells.

## SUMMARY

Being mosaic for the function of their X-linked genes generally ameliorates the expression of X-linked deleterious variants in females. X-inactivation provides the opportunity to share gene products. If this is not possible, then cell selection may eliminate variant cells. Many of these variants affect intellectual ability. Females usually manifest their X-linked pathologic variants, if both alleles are disease variants, or if males with the same pathogenic variants are lost in utero. X-inactivation provides an enormous advantage to females with deleterious X-linked variants, most often enabling them to avoid disease manifestations, including intellectual disability, that affect males. Fortunately, chance skewing that favors mutant alleles is relatively rare (5%) in survivors. The female advantage is reflected by the 20% greater death rate for males at every stage postimplantation, until the age of >75 years, when more females die because there are fewer male survivors.^41^

The females susceptible to X-linked diseases are those who have more than one copy of a pathologic variant, or a relevant second variant, or a variant in an essential gene that does not permit males to survive (Table 2). Females are also susceptible to chance skewing favoring the mutant allele, or the effects of X chromosomal aberrations (i.e., translocations) and monozygotic twinning on inducing unfavorable skewing.^42^

The fact that more males are born than females (1.05:1) is also likely to be attributable to X-inactivation. In this case, preimplantation females are lost because of their greater dosage sensitivity in *maintaining* an active X.^11^ But if XX individuals successfully establish X-inactivation while in utero, then throughout their lifetime, they will benefit from the cellular mosaicism it produces.
